# Supplementary material for: MuRF1 Partners With TRIM72 to Impair Insulin Signaling in Skeletal Muscle Cells
Source: FASEB J. 2025 Sep 26;39(19):e71084. doi: 10.1096/fj.202502066RR (PMC12464895; doi:10.1096/fj.202502066RR)
Supplement: Supplementary file 1 — Figure S1: fsb271084‐sup‐0001‐FigureS1.docx. [file FSB2-39-e71084-s001.docx]

**Supplemental Figure**


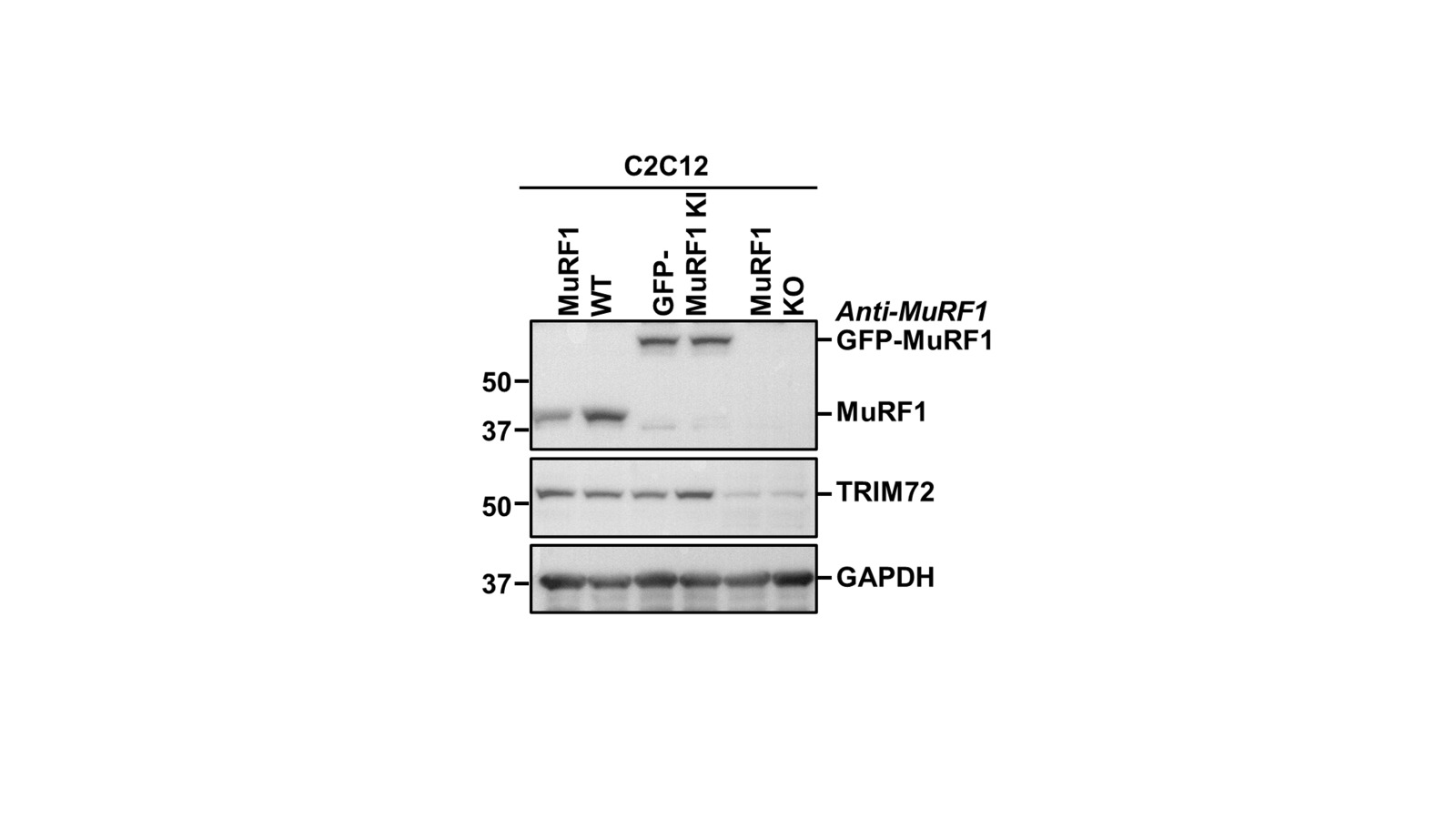


**Figure.S1: TRIM72 abundance remain the same in Wild-type and Crispr/Cas9 generated GFP-MuRF1 knock-in C2C12 myotube.** Cells from Wild type (WT), CRISPR/Cas9-generated GFP-MuRF1 knock-in and C2C12 -MuRF1 KO (as a control) were differentiated into myotubes and lysed before immunoblotting with Anti MuRF1 (A), Anti TRIM72 (B), and Anti GAPDH as a loading control respectively.
